# ALC1/CHD1L, a chromatin-remodeling enzyme, is required for efficient base excision repair

**DOI:** 10.1371/journal.pone.0188320

**Published:** 2017-11-17

**Authors:** Masataka Tsuda, Kosai Cho, Masato Ooka, Naoto Shimizu, Reiko Watanabe, Akira Yasui, Yuka Nakazawa, Tomoo Ogi, Hiroshi Harada, Keli Agama, Jun Nakamura, Ryuta Asada, Haruna Fujiike, Tetsushi Sakuma, Takashi Yamamoto, Junko Murai, Masahiro Hiraoka, Kaoru Koike, Yves Pommier, Shunichi Takeda, Kouji Hirota

**Affiliations:** 1 Department of Radiation Genetics, Graduate School of Medicine, Kyoto University, Yoshidakonoe, Sakyo-ku, Kyoto, Japan; 2 Department of Primary Care and Emergency Medicine, Kyoto University Graduate School of Medicine, Sakyo-ku, Kyoto, Japan; 3 Department of Chemistry, Tokyo Metropolitan University, Minami-Osawa, Hachioji- shi, Tokyo, Japan; 4 Division of Dynamic Proteome, Institute of Development, Aging and Cancer, Tohoku University, Seiryomachi 4–1, Aobaku, Sendai, Japan; 5 Department of Genome Repair, Atomic Bomb Disease Institute, Nagasaki University Sakamoto, Nagasaki, Japan; 6 Department of Genetics, Research Institute of Environmental Medicine (RIeM), Nagoya University, Furo-cho, Chikusa-ku, Nagoya, Japan; 7 Laboratory of Cancer Cell Biology, Radiation Biology Center, Kyoto University, Yoshidakonoe, Sakyo-ku, Kyoto, Japan; 8 Laboratory of Molecular Pharmacology, Developmental Therapeutics Branch, Center for Cancer Research, National Cancer Institute, National Institutes of Health, Bethesda, MD, United States of America; 9 Department of Environmental Sciences and Engineering, University of North Carolina Chapel Hill, North Carolina, United States of America; 10 Department of Mathematical and Life Sciences, Graduate School of Science, Hiroshima University, Hiroshima, Japan; 11 Department of Radiation Oncology, Japanese Red Cross Society Wakayama Medical Center, Komatsubara-Dori, Wakayama, Japan; University of South Alabama Mitchell Cancer Institute, UNITED STATES

## Abstract

ALC1/CHD1L is a member of the SNF2 superfamily of ATPases carrying a macrodomain that binds poly(ADP-ribose). Poly(ADP-ribose) polymerase (PARP) 1 and 2 synthesize poly(ADP-ribose) at DNA-strand cleavage sites, promoting base excision repair (BER). Although depletion of ALC1 causes increased sensitivity to various DNA-damaging agents (H_2_O_2_, UV, and phleomycin), the role played by ALC1 in BER has not yet been established. To explore this role, as well as the role of ALC1’s ATPase activity in BER, we disrupted the *ALC1* gene and inserted the ATPase-dead (E165Q) mutation into the *ALC1* gene in chicken DT40 cells, which do not express PARP2. The resulting *ALC1*^*-/-*^ and *ALC1*^*-/E165Q*^ cells displayed an indistinguishable hypersensitivity to methylmethane sulfonate (MMS), an alkylating agent, and to H_2_O_2_, indicating that ATPase plays an essential role in the DNA-damage response. *PARP1*^*-/-*^ and *ALC1*^*-/-*^*/PARP1*^*-/-*^ cells exhibited a very similar sensitivity to MMS, suggesting that ALC1 and PARP1 collaborate in BER. Following pulse-exposure to H_2_O_2_, *PARP1*^*-/-*^ and *ALC1*^*-/-*^*/PARP1*^*-/-*^ cells showed similarly delayed kinetics in the repair of single-strand breaks, which arise as BER intermediates. To ascertain ALC1’s role in BER in mammalian cells, we disrupted the *ALC1* gene in human TK6 cells. Following exposure to MMS and to H_2_O_2_, the *ALC1*^*-/-*^ TK6 cell line showed a delay in single-strand-break repair. We therefore conclude that ALC1 plays a role in BER. Following exposure to H_2_O_2,_ *ALC1*^*-/-*^ cells showed compromised chromatin relaxation. We thus propose that ALC1 is a unique BER factor that functions in a chromatin context, most likely as a chromatin-remodeling enzyme.

## Introduction

Base excision repair (BER) eliminates nucleotides damaged by oxidation, alkylation, and hydrolysis. There are complex variations within the BER process (for review, see references [1, 2]). A typical BER is initiated by enzymatic removal of the damaged base, leading to the formation of apurinic/apyrimidinic (AP) sites, followed by incision of the DNA backbone at the AP sites, yielding single-strand breaks (SSBs). Since SSB repair and BER share a number of repair factors, such as Poly(ADP-ribose) polymerase (PARP) 1 and 2, SSB repair is considered a specialized BER sub-pathway [3]. PARP1 and PARP2 accumulate quickly at SSB sites, and PARylate themselves as well as chromatin proteins. Poly(ADP-ribose) (PAR) facilitates the recruitment of x-ray-repair cross-complementing group 1 (XRCC1) [4]. XRCC1 plays a key role in SSB repair by providing docking sites for critical effector molecules, polynucleotide kinase 3’-phosphatase (PNKP), DNA polymerase β (Polβ), and ligase 3. PNKP and Polβ restore hydroxyl and phosphorylation residues at the 3’ and 5’ ends, respectively, of the SSBs. Polβ incorporates a single nucleotide, a process called short-patch repair synthesis, for subsequent ligation of SSBs. Polβ, Polδ, and Polθ, on the other hand, undergo long-patch repair synthesis, involving 2–12 nucleotide incorporation, by strand-displacement synthesis, generating a 5’ flap. The Fen-1 endonuclease removes the 5’ flap for subsequent ligation.

Recent studies indicate that PAR is recognized by ALC1 (amplified in liver cancer 1, also known as CHD1L [chromodomain-helicase-DNA-binding protein 1-like]) at its carboxy terminal [5, 6]. ALC1 is a member of the SNF2 superfamily of ATPases, which can function as chromatin-remodeling enzymes [7–9]. ALC1 is believed to play multiple roles in various DNA-damage responses, since depletion of ALC1 causes hypersensitivity to UV, H_2_O_2_, and phleomycin [6, 10], which induce lesions repaired primarily by nucleotide excision repair, BER, and double-strand break (DSB) repair, respectively. The role played by PARP in both the SSB- and DSB-repair pathways, and the conversion of SSBs to DSBs during DNA replication, make the hypersensitivity of ALC1-depleted cells very difficult to interpret. Another complexity of ALC1’s role is involvement of ALC1 in transcriptional control for effective DNA-damage responses, as evidenced by the observation that ALC1 interacts with Tripartite Motif-containing 33 (TRIM33), a multifunctional protein implicated in transcriptional regulation [11]. Collectively, whether or not ALC1 promotes BER has remained elusive, and the functional relationship between ALC1 and PARP1 has been also undefined.

We disrupted the *ALC1* gene in the human TK6 and chicken DT40 B cell lines. We also disrupted the *ALC1* gene in PARP1-deficient DT40 cells. DT40 has a unique advantage for reverse-genetic study of the PARP enzyme due to the absence of the *PARP2* gene in the chicken genome [12]. Note that the chicken *XRCC1* ortholog gene has not yet been identified. PARP1-deficient *PARP1*^*-/-*^ DT40 cells show an increased methylmethane sulfonate (MMS) sensitivity and a marked accumulation of SSBs [13]. We found that *ALC1*^*-/-*^, *PARP1*^*-/-*^, and *ALC1*^*-/-*^*/PARP1*^*-/-*^ DT40 cells were markedly sensitive to both H_2_O_2_ and MMS, suggesting that ALC1 collaborates with PARP1-medaited BER. We examined the role played by ALC1 in BER by conducting alkaline-comet and alkaline-elusion assays, which are the established methods of monitoring ongoing BER [14, 15]. Our data demonstrate that ALC1 promotes BER in both TK6 and DT40 cells. We conclude that ALC1 plays a critical role in BER, under the control of PARP1.

## Materials and methods

### DT40-cell culture, cell counting, and cell-cycle analysis

DT40 cell line was from Takeda laboratory (Kyoto University) [16]. Culture conditions for the chicken DT40 cells, cell counting, and cell-cycle analysis were as described previously [17–20].

### TK6-cell culture

TK6 cell line was purchased from JCRB cell bank (http://cellbank.nibiohn.go.jp/english/). TK6 cells [21] were cultured in an RPMI 1640 medium (Nacalai Tesque, Kyoto, Japan) supplemented with 10% heat-inactivated horse serum (HS) (GIBCO, lot No. 2017–06), 0.1 mM sodium pyruvate, L-glutamin (Nacalai Tesque), 50 U/mL penicillin, and 50 μg/mL streptomycin (Nacalai Tesque). TK6 has a stable, near-diploid karyotype, though it carries a trisomic chromosome 13 [22].

### si-RNA treatment

HeLa and U2OS cells were cultured in DMEM supplemented with 10% fetal-calf serum at 37° C. si-RNAs for the depletion of ALC1 and the control were purchased from Thermo Scientific (Dhamacon si-RNA, PA). 3 × 10^5^ cells were transfected with 250 pmol si-RNA using 10μl Lipofectamine RNAiMAX (Invitrogen, CA).

### Genotoxic reagents

H_2_O_2_ (Nacalai) and MMS (Nacalai) were used for the sensitivity assay, as described previously [23].

### Measurement of sensitivity to genotoxic agents in DT40 and TK6 cells

10^6^ DT40 cells were exposed to H_2_O_2_ and MMS for 1 h in 1 ml culture medium and PBS containing 1% fetal bovine serum, respectively. TK6 cells were exposed to the two DNA-damaging agents in the same manner. 0.01 ml cell suspensions (containing 10^4^ cells) of the exposed DT40 or TK6 cells were plated in duplicate onto 24-well cluster plates containing 1 ml of the complete medium, and were incubated for 48 h (for DT40) and 72 h (for TK6). To measure the number of living cells, we transferred 100 μl cell suspensions to the individual wells of 96-well plates and measured the amount of ATP in the cellular lysates using CellTiter-Glo (Promega), according to the manufacturer's instructions. Luminescence was measured with Fluoroskan Ascent FL (Thermo Fisher Scientific Inc., Pittsburgh, PA).

### Generation of *ALC1* targeting constructs for DT40 cells

*ALC1* targeting constructs were generated from genomic PCR products combined with *BSR*^R^ or *HIS*^*R*^ selection-marker cassettes. Genomic DNA sequences were amplified using primers 5'-CCGGAATTCTTACACCCAGGCACACAAAA-3' and 5'-CGCGGATCCCTGGAAGCCACCAGAAGAAG-3' (for the left arm), and 5'-CGCGGATCCCATGGCAGTGAAACATGGAC-3' and 5'-ATTGCGGCCGCCTGCAATGCAAAAGACCTGA-3' (for the right arm). The resulting amplified fragments were inserted into the *Eco*RI and *Not*I sites of pBluescript II (Stratagene). Marker-gene cassettes, *BSR*^R^ and *HIS*^R^, were then inserted into the *Bam*HI site of the resulting plasmid to generate *ALC1-BER* and *ALC1-HIS* gene-targeting constructs. A probe for Southern blot was amplified from DT40 genomic DNA using primers 5'-AAACGTTACGCCTTAGGCTCGTTGCTTCTT-3' and 5'-CATTCTGTGATTCTATGTTTTCAGCTTC-3'.

### Generation of *ALC1*^*-/-*^ and *ALC1*^*-/-*^*/PARP1*^*-/-*^ mutant DT40 cells

We sequentially transfected *ALC1-BSR-* and then *ALC1-HIS*-targeting constructs to obtain *ALC1*^-/-^ cells from *wild-type* DT40 cells. Similarly, we generated *ALC1*^*-/-*^*/PARP1*^*-/-*^ mutant clones from *PARP1*^*-/-*^ cells [24].

### Generation of *ALC1*^*-/E165Q*^ mutant DT40 cells

To selectively inactivate the ATPase activity of ALC1, we generated an E165Q mutation knock-in construct carrying the *HIS*^R^ selection-marker cassette. Genomic DNA sequences were amplified using primers 5'-TGAGCAACTGGAAGGAGGAGCTGGAGAG-3' and 5'-AAAAGTCTTTATACAGTACAAGATAGCTGG-3' (for the left arm), and 5'-GGGATCCCACTATAGGATACAGATTTTCTGTTTATTC-3' and 5'- TTCTAGAACTTACCTGGCTCACTCTCCTTTTCAACTG-3' (for the right arm). The amplified fragment for the left arm was mutated using primers 5’-GCCTTGGTTGTAGATCAAGCTCACAGGCTG-3’ and 5’-CAGCCTGTGAGCTTGATCTACAACCAAGGC-3’ (the E to Q mutation is associated with the underlined *Sau*3AI site). The obtained left arm and the right arm were cloned into Zero Blunt TOPO (Invitrogen, CA). The 3.9 kb *Xho*I/*Bam*HI fragment from the left arm and the 1.4 kb *Bam*HI/*Xba*I fragment from the right arm were inserted into the *Xho*I/*Xba*I site of pBluescript vector (Stratagene). A marker-gene cassette, loxP-flanked *HIS*^R^, was then inserted into the *Bam*HI site of the resulting plasmid to generate the E165Q mutation knock-in construct. The *ALC1*^*-/+*^ cells were then transfected with the E165Q mutation knock-in construct to obtain *ALC1*^*-/E165Q*^ cells. Targeted integration into the *wild-type* allele was confirmed by Southern blot analysis of *Bam*HI-digested genomic DNA, with the use of an internal probe prepared from a 1.0 kb *Eco*RI fragment in the left arm using a Random Primer kit (GE Healthcare, UK). To delete the loxP-flanked *HIS*^R^ cassette, a Cre expression vector was transiently transfected using *Amaxa*™ Nucleofector™ 2b (Lonza) and then incubated for 48 h in medium containing tamoxifen. We performed limiting dilution of cells and isolated clones. RT-PCR was then performed for individual clones, using primers 5'-CCTGATACTTTGTCCTCTGTCTGTTCTGAG-3' and 5'-TCCAATCTCAAAGGGCTCCGGCTCAACACC-3'. The knock-in mutation was screened by identifying 0.24 kb + 0.49 kb *Sau*3AI-digested fragments in the amplified PCR product, and was verified by nucleotide sequencing.

### Generation of *ALC1*^*-/-*^ mutant TK6 cells

*ALC1* targeting vector was constructed to replace part of the 8th exon with a resistance (*Puro* and *Neo*)-gene cassette flanked by loxP signals at both ends. The primers used to amplify the left arm were 5’-CCTCGAGCTCAGTAGTCTTCAGTCTCCTGTTGAC-3’ and 5’-GGCTAGCGCTGCAAGAGTTTGTGCAGTTCACTTG-3’, and the primers for the right arm were 5’-GGCGGCCGCGCATTGCAGAAGAAATACTACAAGGCC-3’ and 5’-GGTCGACATATGGGTGATCCACACACTTTCGAAG. Expression vector for transcription activator-like effector nuclease (TALEN) was designed to recognize the following sequences according to the method described in Sakuma *et al* [25].: 5’-TGCACAAACTCTTGCAG-3’ and 5’-CGAGTGAAAGCTGAGGTA-3’. To generate *ALC1*^*-/-*^ cells, *wild-type* TK6 cells were transfected with the *ALC1* targeting vectors (*Puro*^*R*^ and *Neo*^*R*^) with expression vector for TALEN using NEON transfection system (Invitrogen, CA) at 1500 V 20 msec. The loss of *ALC1* transcript was confirmed by RT-PCR using primers 5’- CAAGAAGACA GAAGTAGTGA TATACCATGG-3’ and 5’- CCATATAGTCTTGGAGAATATCCAACATCT-3’. *GAPDH* transcripts were analyzed as a positive control for the RT-PCR analysis using primers 5’- TGGCCAAGGTCATCCATGACAACTT-3’ and 5’- GCGCCAGTAGAGGCAGGGATGATGT -3’. The loss of ALC1 protein was confirmed by western blot using anti-ALC1 antibody (abcam, ab51324). β-actin was detected using specific antibody (Sigma, A5441) as a loading control.

### Determination of intracellular NAD(P)H concentration

The cellular NAD(P)H concentration was determined as previously described [26].

### Detection of GFP-XRCC1 at the site of SSBs induced by *Neurospora crassa* UV-damage endonuclease (UVDE)

Local UV irradiation and detection of GFP-XRCC1 with immunofluorescence microscopy was conducted as previously described [27]. Cyclobutane pyrimidine dimer (CPD) induced by UV was detected using anti-CPD polyclonal antibody.

### Partial digestion of chromatin DNA with micrococcal nuclease (MNase)

Partial digestion of chromatin DNA with MNase was performed as described previously [28–30]. Briefly, 5×10^7^ cells were harvested and suspended well by pipetting in 0.5 ml of lysis buffer (18% Ficoll 400, 10 mM KH_2_PO_4_, 10 mM K_2_HPO_4_, 1 mM MgCl_2_, 0.25 mM EGTA, 0.25 mM EDTA, and complete^™^ Protease Inhibitor Cocktail [Roche, Mannheim, Germany]). After centrifugation at 14,000 rpm for 30 min at 4°C, the crude nuclear pellet was resuspended in 0.6 ml of buffer A (10 mM Tris-HCl [pH 8.0], 150 mM NaCl, 5 mM KCl, 1 mM EDTA, and 1 mM Pefabloc SC [Roche, Mannheim, Germany]). After addition of CaCl_2_ (5 mM final concentration), 0.1 ml aliquots of crude nuclear suspension were digested with different concentration of MNase (0, 10, and 20 U/ml) at 37°C for 5 min. The reaction was terminated by adding 25 mM EDTA, and the DNA was purified by incubation with 1% SDS and 20 μg/ml proteinase K (Merck, Damstadt, Germany) at 55°C, followed by phenol-chloroform extraction.

### Isolation of nuclear-soluble and chromatin-bound fractions from DT40 cells

We isolated the nuclear soluble fraction from DT40 cells using the Subcellular Protein Fractionation Kit for Cultured Cells (Thermo, PA). Histone and Topoisimerase I was detected using following specific antibodies (anti-Histone H3 from MBL, MABI0301 and anti-Topoisomerase I from BD Pharmingen^TM^, 556597).

### Alkaline comet assay

For the chicken DT40 cells, the tail DNA percentage, reflecting the number of SSBs (% DNA in tail) [31], was measured for cells that had been exposed to 25 μM H_2_O_2_ for 20 min on ice, and cells that had been exposed to 25 μM H_2_O_2_ for 20 min on ice followed by a 30 min repair period at 39.5°C. For DT40 cells, a Comet Analysis System was used to quantify the comet tails (Komet 3.0, Kinetic Imaging Ltd., Liverpool, UK). Human TK6 cells were treated with 80 μM H_2_O_2_ on ice for 30 min or with MMS at 37°C for 15 min and subsequently released in drug-free, pre-warmed culture medium. Alkaline-comet and single-cell gel electrophoresis assays were performed as described previously [14, 15, 32]. Electrophoresis was carried out by applying 25 volts at 4°C for 50 min using a submarine gel electrophoresis machine (Cat. NB-1012, NIHON EIDO CO. Ltd.) filled with 1850 ml running buffer (0.3 M NaOH, 1 mM EDTA). For TK6 cells, a Comet Analysis System was used to quantify the comet tails (Comet analyzer, YOUWORKS CO. Ltd.). 100 cells were scored per sample.

### Alkaline elution assay

The number of SSBs was quantified using the alkaline elution assay, as described previously [33, 34]. Chicken DT40 cells were radiolabeled with [^14^C] thymidine (0.01–0.02 μCi/ml) for 16 h, chased in radioisotope-free medium for 4 h, followed by H_2_O_2_ treatment. Alkaline elution was then carried out under deproteinizing, DNA-denaturing conditions, and fractions were collected into scintillation vials at 180 min intervals. Radioactivity in each fraction was analyzed with a liquid scintillation analyzer (Packard Instruments, Meridien, CT).

## Results

### *ALC1*^*-/-*^ DT40 cells are hypersensitive to H_2_O_2_ and MMS

**Fig 1:**
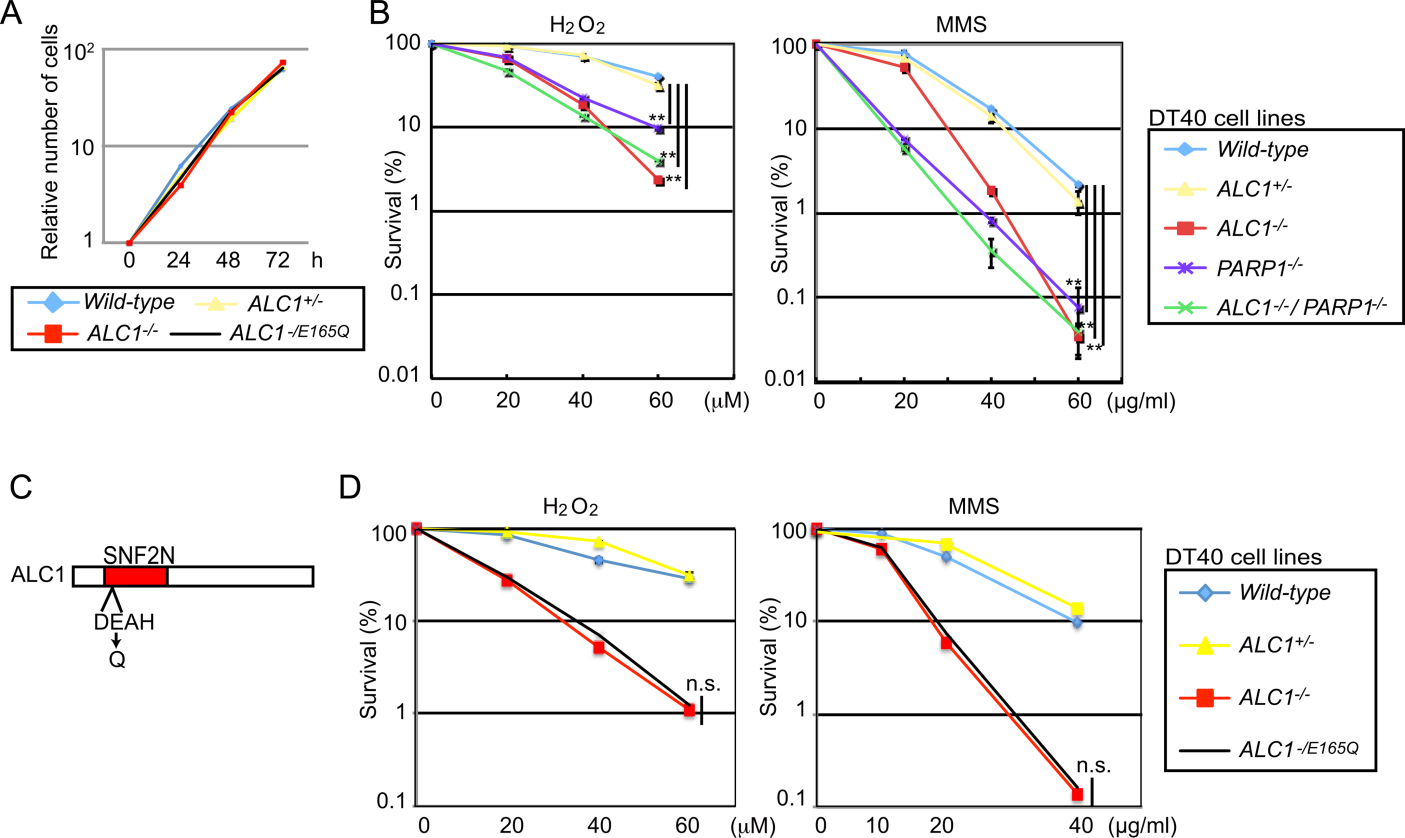
The *ALC1*^*-/-*^ mutation is epistatic to the *PARP1*^*-/-*^ mutation in DT40-cell sensitivity to H_2_O_2_ and MMS. (A) Growth curves corresponding to the indicated cell cultures. (B) DT40 cells of the indicated genotypes were exposed to H_2_O_2_ and methyl methanesulfonate (MMS). The dose of the genotoxic agent is displayed on the x-axis on a linear scale, while the percent fraction of surviving cells is displayed on the y-axis on a logarithmic scale. Error bars represent standard deviations for more than three experiments. (C) Schematic of the SNF2N domain in ALC1. The amino acid change in *ALC1*^*-/E165Q*^ cells is shown below. (D) The sensitivity of *ALC1*^*-/-*^ and *ALC1*^*-/E165Q*^ DT40 cells to H_2_O_2_ and MMS was very similar. Data as in B. *p*-value was calculated by a student’s *t*-test: *p* (**) <0.01 and n.s. (not significant).

We disrupted the *ALC1* gene in the chicken DT40 line (panel A, B in S1 Fig) and found that the resulting *ALC1*^*-/-*^ cells proliferated with normal kinetics (Fig 1A) and showed a normal cell-cycle distribution (panel C in S1 Fig). To investigate the role played by ALC1 in BER, we measured sensitivity to H_2_O_2_ and MMS [35, 36]. The *ALC1*^*-/-*^ cells were hypersensitive to H_2_O_2_ and MMS, as were the *PARP1*^*-/-*^ cells (Fig 1B). To learn more about ALC1 as a chromatin-remodeling factor, we inactivated ATPase activity by mutating the essential E165 to Q (the equivalent mutation used in [6, 37]) of the endogenous *wild-type ALC1* allelic gene in *ALC1*^*-/+*^ cells (Fig 1C and S1D Fig). The resulting *ALC1*^*-/E165Q*^ and *ALC1*^*-/-*^ cells displayed virtually the same phenotype (Fig 1A and 1D), suggesting that ALC1 may promote DNA repair as a chromatin remodeler.

### Epistatic relationship between ALC1 and PARP1 in cellular tolerance to H_2_O_2_ and MMS

To analyze the functional relationship between ALC1 and PARP1, we disrupted the *ALC1* gene in *PARP1*^*-/-*^ DT40 cells, generating *ALC1*^*-/-*^*/PARP1*^*-/-*^ cells. We then analyzed cellular sensitivity to H_2_O_2_ and MMS by comparing *wild-type*, *PARP1*^*-/-*^, *ALC1*^*-/-*^, and *ALC1*^*-/-*^*/PARP1*^*-/-*^ clones.

While sensitivity to H_2_O_2_ was higher in the *ALC1*^*-/-*^ DT40 cells than in the *PARP1*^*-/-*^ clones, it was very similar in the *ALC1*^*-/-*^ and *ALC1*^*-/-*^*/PARP1*^*-/-*^ clones (Fig 1B), indicating an epistatic relationship between ALC1 and PARP1. The *ALC1*^*-/-*^ cells, on the other hand, were slightly less sensitive to MMS than were the *PARP1*^*-/-*^ clones. Nonetheless, ALC1 and PARP1 do have an epistatic relationship, as sensitivity to MMS was very similar for the *PARP1*^*-/-*^ and *ALC1*^*-/-*^*/PARP1*^*-/-*^ clones (Fig 1B). These observations indicate that ALC1’s role in cellular tolerance to H_2_O_2_ and MMS depends at least partially on the functionality of PARP1. In conclusion, given that PARP1 contributes to H_2_O_2_ and MMS tolerance by promoting BER, ALC1 may play a role in BER in the chicken DT40 cell line.

### The important role of ALC1 in BER

**Fig 2:**
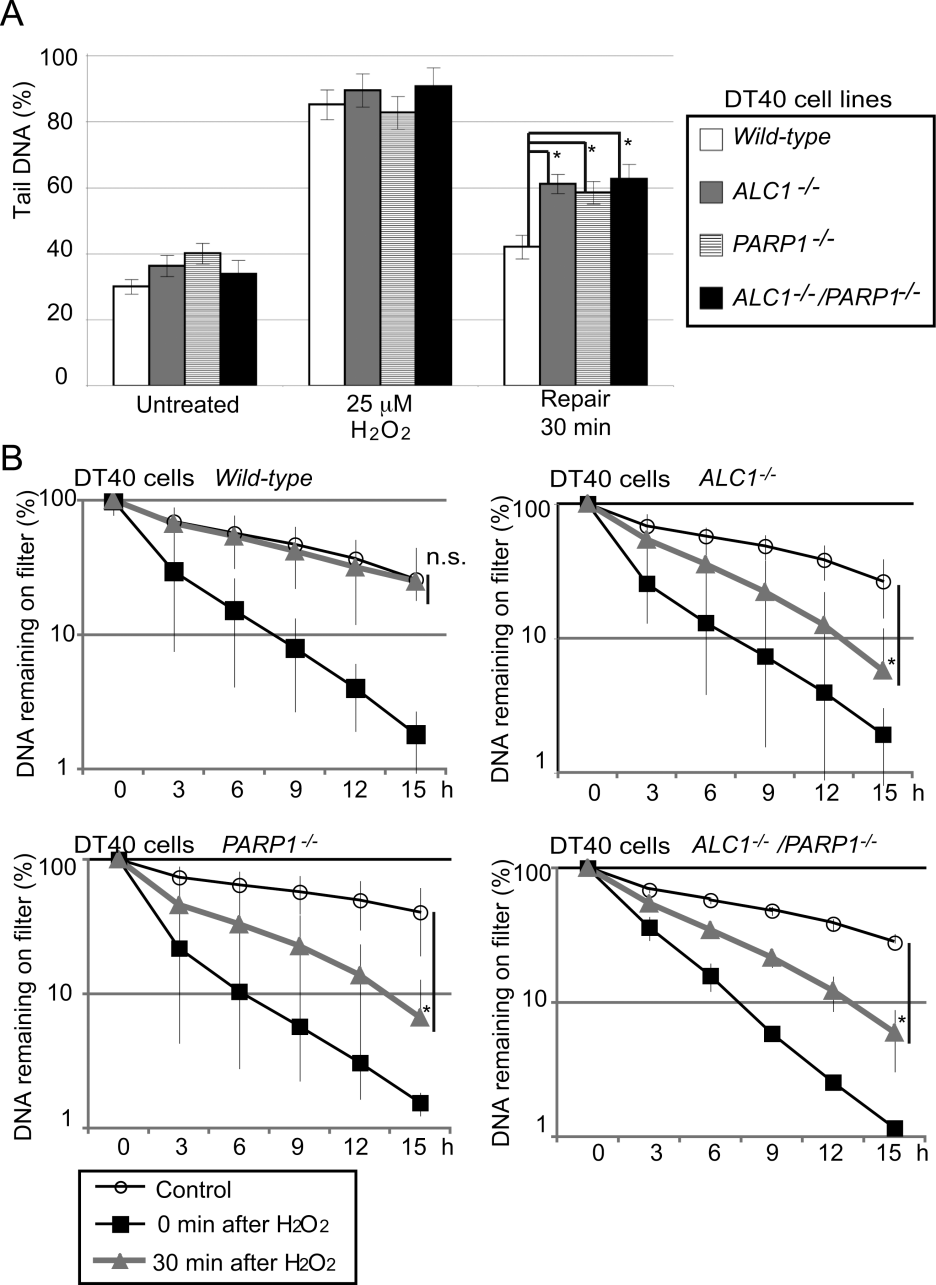
ALC1 is required for the repair of SSBs, which are BER intermediates, in DT40 cells. (A) Alkaline-comet assay to detect SSBs. Indicated genotype were tested in untreated cells, immediately after exposure to 25 μM H_2_O_2_ on ice, and after a further 30 min incubation (repair period). Error bars represent standard deviations from three independent experiments. Tail-DNA percentage, defined as percentage of damaged DNA (% DNA in tail), was calculated as described in Materials and Methods. Tail-DNA % is displayed on the y-axis on a linear scale. (B) Alkaline elution experiment to detect SSB frequency. The data are expressed as the fraction of DNA remaining on the filter for the indicated genotypes of untreated cells (white circle), pulse H_2_O_2_-treated cells (black square), and the H_2_O_2_-treated cells incubated for a further 30 min (gray triangle). Elution time is displayed on the x-axis on a linear scale. The percentage of DNA remaining on the filter is displayed on the y-axis on a logarithmic scale. Error bars represent standard deviations from three independent experiments. *p*-value was calculated by a student’s *t*-test: *p* (*) <0.05, and n.s. (not significant).

The above data suggest that ALC1 may facilitate BER. We thus monitored ongoing BER by measuring the number of SSBs, which are BER intermediates, after a 20 min H_2_O_2_ treatment on ice [14, 15]. We then performed the alkaline-comet (Fig 2A) and alkaline-elusion assays (Fig 2B). Immediately after H_2_O_2_ pulse-treatment, tail sizes in *wild-type* and *ALC1*^*-/-*^ cells were very similar (Fig 2A). At 30 min after treatment, tails in the *wild-type* cells had decreased to nearly normal size, while tails in the *ALC1*^*-/-*^ cells were significantly longer. Likewise, alkaline elusion showed that 30 min after treatment, *wild-type*, but not *ALC1*^*-/-*^ cells, had eliminated the vast majority of SSBs (Fig 2B). Importantly, the *ALC1*^*-/-*^, *PARP1*^*-/-*^, and *ALC1*^*-/-*^*/PARP1*^*-/-*^ clones all showed similar kinetics in SSB repair in both assays (Fig 2A and 2B). These data demonstrate that ALC1 and PARP1 collaborate in SSB repair in chicken DT40 cells.

**Fig 3:**
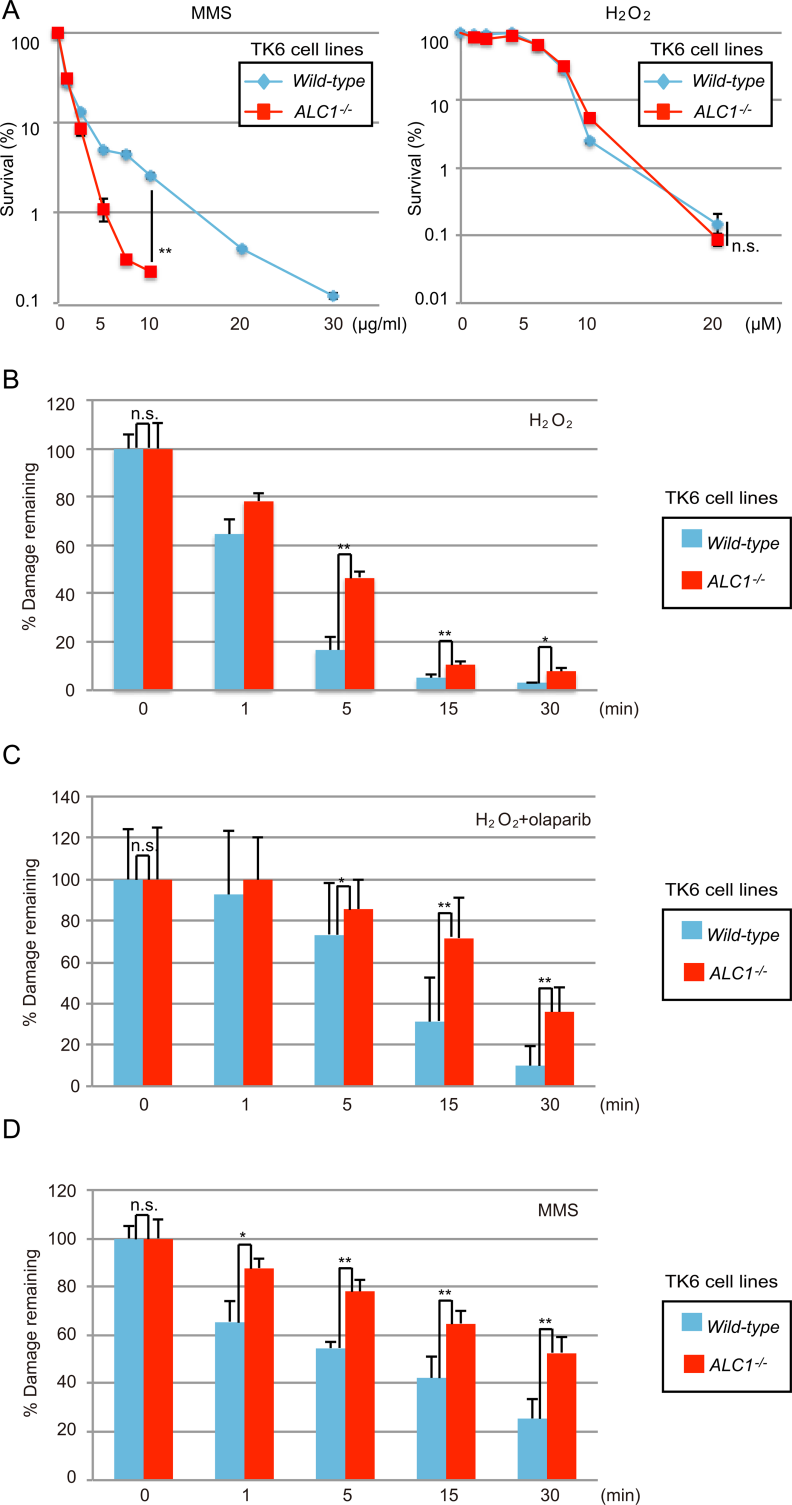
ALC1 is required for the repair of SSBs, which are BER intermediates, in human TK6 cells. (A) DNA-damage sensitivity of TK6 cells carrying the indicated genotype. Data as in Fig 1B. (B–D) Alkaline-comet assay to detect unrepaired SSBs in human TK6 cells. The percentage of DNA strand breaks remaining at the indicated time points is displayed on the y-axis on a linear scale. Error bars represent standard deviations from three independent experiments. (B) Human TK6 cells of the indicated genotype were exposed to 80 μM H_2_O_2_ on ice for 30 min. The cells were released in drug-free, pre-warmed culture medium and further cultured for the indicated time. (C) TK6 cells treated with H_2_O_2_ as in B were released in culture medium containing 1 μM olaparib then cultured for the indicated time. (D) *Wild-type* and *ALC1*^-/-^ TK6 cells were treated with 0.1 and 0.075 mg/ml MMS, respectively, for 15 min. Cells were then released in drug-free, pre-warmed culture medium and further cultured for indicated time. *p*-value was calculated by a student’s *t*-test: *p* (**) <0.01, (*) <0.05, and n.s. (not significant).

To examine ALC1’s role in BER in human cells, we created *ALC1*^-/-^ clones from the human TK6 cell line (S2 Fig) and analyzed SSB-repair kinetics by performing an alkaline-comet assay in *wild-type* and *ALC1*^-/-^ TK6 cells. The *ALC1*^-/-^ TK6 cells were more sensitive to MMS than were the *wild-type* cells (Fig 3A). Unlike the DT40 cells, the *ALC1*^-/-^ TK6 cells showed similar H_2_O_2_ sensitivity compared to *wild-type* (Fig 3A). It is possible that the oxidative stress decreased cellular viability through mechanisms other than DNA damage in this cell line. Nonetheless, delayed SSB repair at 5 min following pulse-exposure to H_2_O_2_ was reproducibly seen in the *ALC1*^*-/-*^ TK6 cells (Fig 3B). Moreover, when the pulse-exposed cells were subsequently treated with the chemotherapeutic PARP poison, olaparib, which stabilizes the PARP-DNA complex, the delay in SSB repair in the *ALC1*^*-/-*^ TK6 cells was more pronounced than in the *wild-type* cells [38] (Fig 3C). We thus conclude that ALC1 contributes to SSB repair in the human cell line as well as in the chicken DT40 cells. We examined the kinetics of BER following pulse-exposure to MMS. Note that pulse-exposure to MMS has to be done at 37°C, at which temperature base damage and repair occur in parallel. Alkaline-comet tails were longer in *ALC1*^*-/-*^ than in *wild-type* cells after pulse-exposure to MMS (S3 Fig), suggesting a BER defect in the *ALC1*^*-/-*^ cells. To examine the actual BER kinetics, we pulse-exposed *wild-type* and *ALC1*^-/-^ cells to MMS and released in drug-free medium. Repair in the *ALC1*^*-/-*^ cells was significantly delayed during the chase period (Fig 3D). We thus conclude that ALC1 promotes BER in both chicken DT40 and human TK6 cell lines.

### ALC1 is dispensable for the recruitment of XRCC1 to damage sites

**Fig 4:**
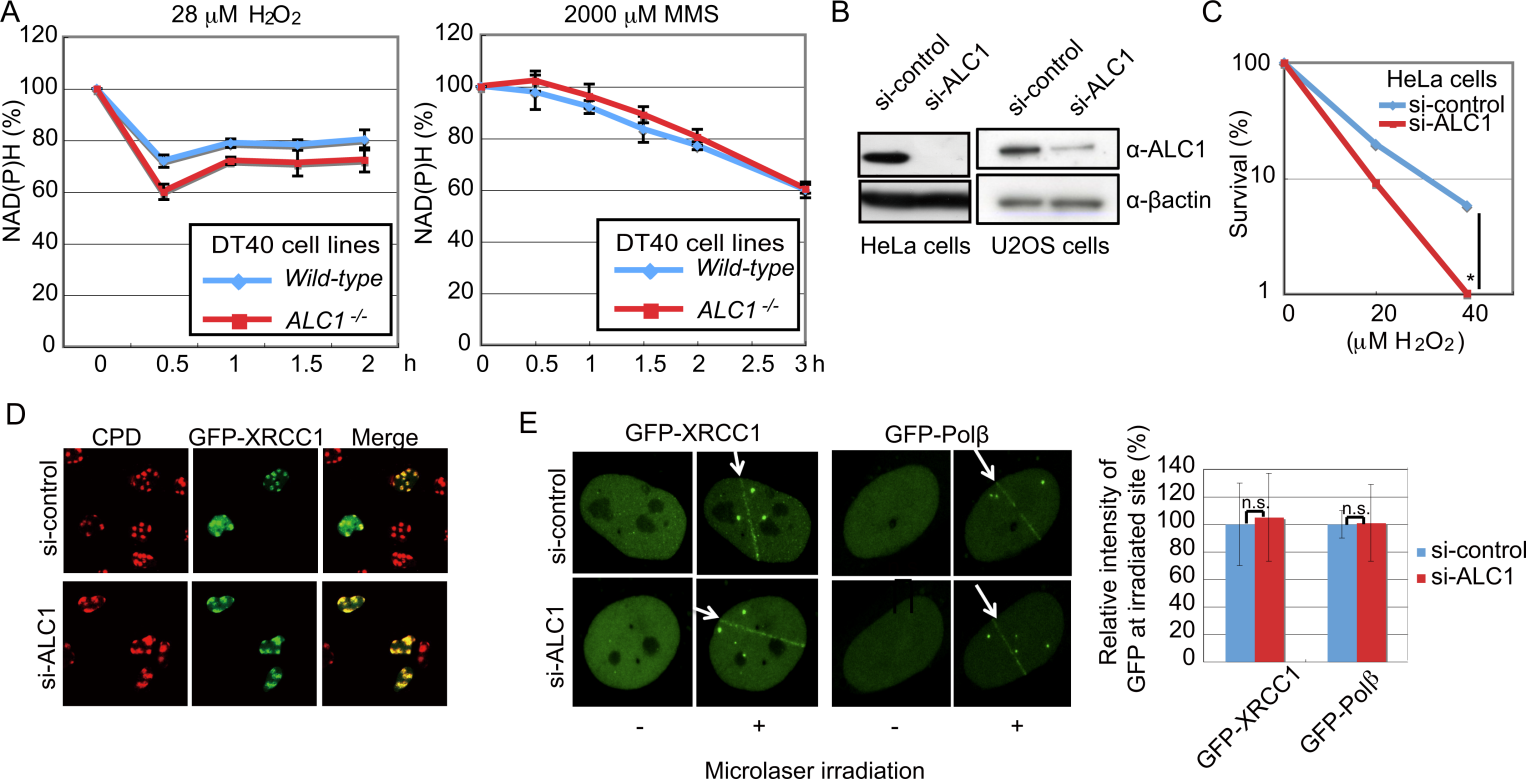
ALC1 is not required for PARylation or recruitment of Polβ or XRCC1 to DNA-damage sites. (A) DT40 clones of the indicated genotypes were treated with H_2_O_2_ or MMS and harvested at the indicated times after treatment. To evaluate the activation of the PARylation event by PARP1 at SSB sites, we measured the cellular concentration of NAD(P)H, the major substrate for PARP. Time after treatment with H_2_O_2_ or MMS is displayed on the x-axis, while the relative cellular NAD(P)H concentration is displayed on the y-axis. (B) Knockdown of ALC1 using si-RNA in human HeLa and U2OS cells was verified by western blot analysis using anti-ALC1 and anti-βactin (loading control). (C) H_2_O_2_ sensitivity of ALC1-depleted or control si-RNA-treated HeLa cells. The dose of H_2_O_2_ is displayed on the x-axis on a linear scale, while the percentage of cell survival is displayed on the y-axis on a logarithmic scale. (D) U2OS cells deficient in nucleotide excision repair, xeroderma pigmentosum group A^-^ (XPA^-^) expressing *Neurospora crassa* UV-damage endonuclease (UVDE) were exposed to 100 J/m^2^ UV through micro pores in membrane filters. DNA single-strand breaks were produced at UV-irradiated region, where GFP-XRCC1 accumulated independently of ALC1 expression. Representative image of XPA-UVDE cells displaying GFP-XRCC1 and UV damage (CPD) signals. (E) GFP-XRCC1 and GFP-Polβ accumulate immediately at DNA damage sites after exposure to a 405 nm pulse laser in U2OS cells, which have been treated with siRNA against ALC1 or control siRNA for 48 h as previously described [41]. GFP-signal intensity for GFP-XRCC1 and GFP-Polβ is displayed in the histogram. Error bars represent standard deviations from three independent experiments. *p*-value was calculated by a student’s *t*-test: *p* (*) <0.05, and n.s. (not significant).

Having confirmed that ALC1 and PARP1 have an epistatic relationship in BER in chicken DT40 cells, we next attempted to define the role played by ALC1 in the promotion of BER by PARP. To determine whether or not ALC1 controls PARP-mediated PARylation, we measured intracellular NAD(P)H during continuous exposure to H_2_O_2_ and MMS [26, 27, 39, 40]. This assay monitors PARP-mediated PARylation, since reduction of NAD^+^, a major substrate of PARP, results in depletion of cellular NAD(P)H [26]. We found that the amount of NADPH was reduced in *wild-type* and *ALC1*^*-/-*^ DT40 cells with very similar kinetics (Fig 4A), indicating that PARylation occurs normally in *ALC1*^*-/-*^ cells [38].

We considered that ALC1 might control PARP-mediated recruitment of XRCC1 to damage sites. To test this hypothesis, we induced SSBs selectively in subnuclear areas and examined the kinetics of XRCC1 relocalization to SSB sites [42]. To induce SSBs, we employed a nucleotide-excision, repair-deficient xeroderma pigmentosum group A (XPA) U2OS cells expressing *Neurospora crassa* UV-damage endonuclease (UVDE), which generates SSBs at UV photoproducts [27, 43]. We depleted ALC1 using si-RNA in Hela and U2OS cells (Fig 4B) and monitored the recruitment of BER factors to induced SSB sites after UV irradiation. As with the *ALC1*^*-/-*^ DT40 cells (Fig 1), the ALC1-depleted HeLa cells showed a higher sensitivity to H_2_O_2_ than did the si-control-RNA-treated cells (Fig 4C). On the other hand, the depletion of ALC1 did not impair the recruitment of XRCC1 to induced SSB sites (Fig 4D). Similarly, GFP-XRCC1 was efficiently recruited to laser-induced DNA damage sites in ALC1-depleted and control cells (Fig 4E). Moreover, recruited XRCC1 seemed to be functional, since GFP-Polβ also accumulated at DNA-damage sites (Fig 4E). We thus conclude that ALC1 contributes to BER independently of XRCC1.

### ALC1 facilitates BER by relaxing chromatin at DNA-damage sites

**Fig 5:**
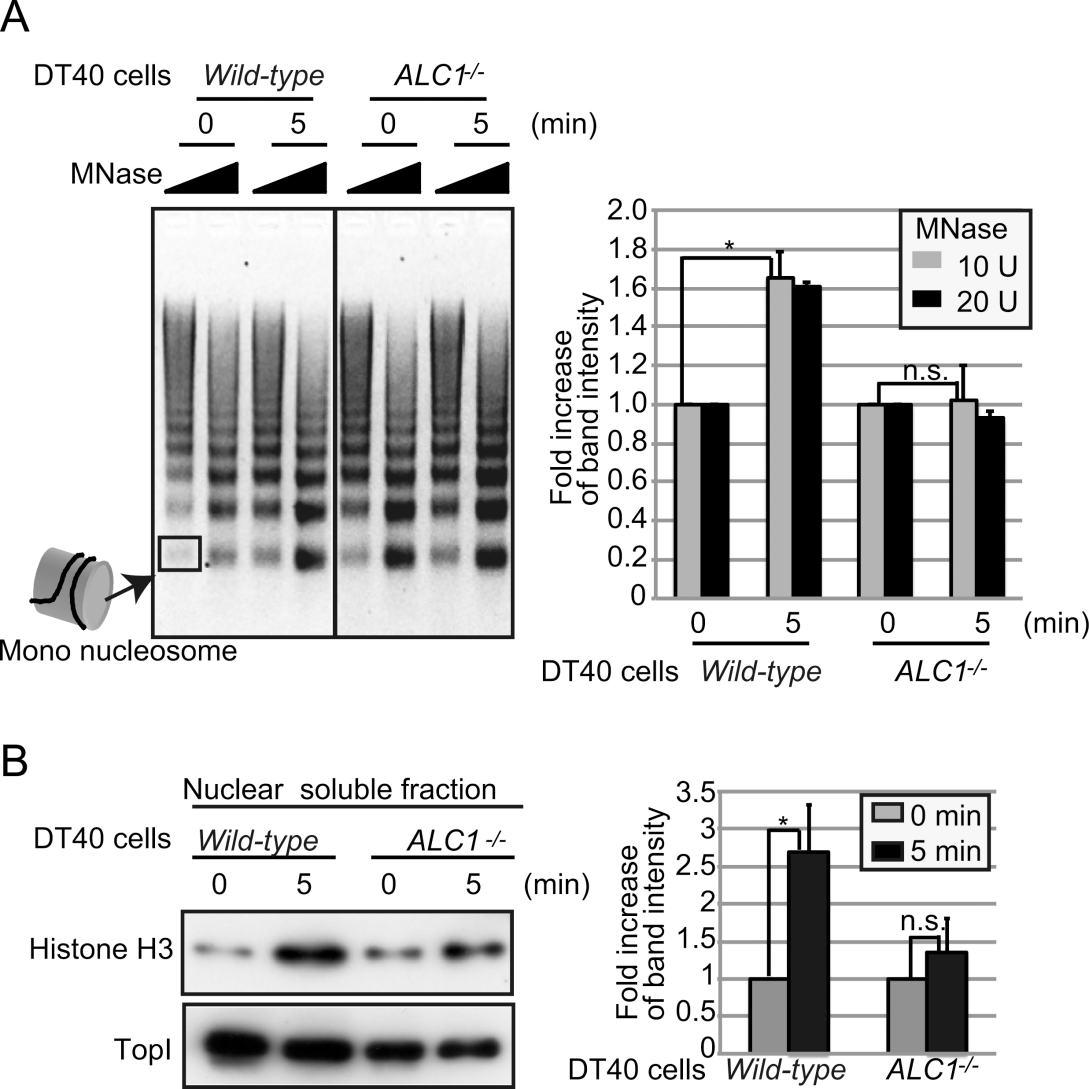
ALC1 facilitates DNA-damage-induced chromatin relaxation. (A) DT40 cells of the indicated genotypes were treated with H_2_O_2_, and cells were harvested at the indicated times after H_2_O_2_ treatment. The chromatin fraction was digested with 10 and 20 U/ml MNase. Digested genomic DNA products were analyzed by gel electrophoresis. The relative intensity of the band corresponding to the mono-nucleosome (146 bp, indicated by arrow) was quantified. (B) Histone H3 present in the nuclear soluble fraction was detected by western blot. The blot was probed with anti-Topoisomerase I (TopI) antibody as a loading control. The relative intensity of the band corresponding to histone H3 was quantified and normalized for the level of TopI. *p*-value was calculated by a student’s *t*-test: *p* (*) <0.05, and n.s. (not significant).

The above conclusion led us to consider that ALC1 might promote chromatin relaxation at DNA-damage sites to facilitate DNA repair. To monitor the extent of chromatin compaction, we performed the MNase chromatin digestion assay. We exposed chicken DT40 cells to H_2_O_2_, then partially digested chromatin DNA with MNase, and quantified the fraction of the mono-nucleosome. Five min after treatment with 5 mM H_2_O_2_, the amount of partially digested product, ~146 bp DNA (Fig 5A, box), was significantly increased in *wild-type* cells (Fig 5A, lanes 1, 2 and 3, 4), but not in *ALC1*^*-/-*^ cells (Fig 5A, lanes 5, 6 and 7, 8). The data suggest that configuration of chromatin changes into open status in *wild-type* but not in *ALC1*^*-/-*^ cells by DNA-damage presumably through inducing histone eviction (Fig 5A). To further examine this possibility, we measured the amount of histone H3 released from the chromatin after DNA damage (Fig 5B). The amount of histone H3 in the nuclear soluble fraction was increased in *wild-type* cells after H_2_O_2_ treatment, suggesting that DNA-damage induces histone eviction from chromatin (Fig 5B). However, such histone eviction was reduced in *ALC1*^*-/-*^ cells (Fig 5B). These results suggest that ALC1 promotes BER by inducing chromatin remodeling through histone eviction and facilitating the access of BER factors to DNA-damage sites.

### The *ALC1* locus is unstable and susceptible to gene amplification in a large number of malignant tumors

**Fig 6:**
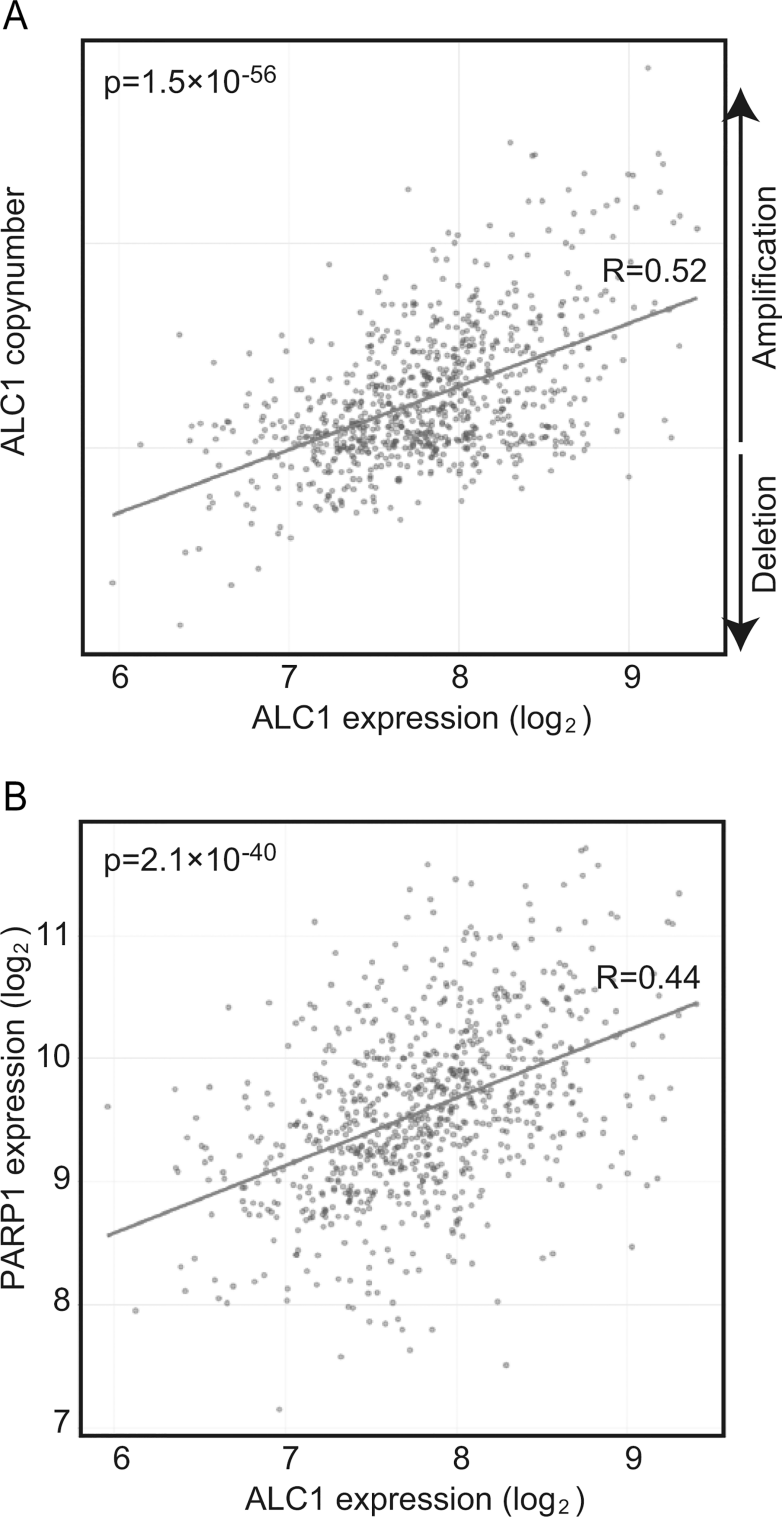
Overexpression of *ALC1* in a wide variety of cancer cell lines. (A) Transcriptional characteristics of ALC1 were surveyed in 1000 cancer-cell lines using the Cancer Cell Line Encyclopedia [44]. The relative expression level of *ALC1* in the cancer-cell lines is displayed on the x-axis on a logarithmic scale, while the relative copy number of the *ALC1* gene is displayed on the y-axis on a linear scale. (B) Relationship between *ALC1* and *PARP1* expression levels in 1000 cancer cell lines. The relative expression of *ALC1* is displayed on the x-axis, while the relative expression of *PARP1* is displayed on the y-axis on a logarithmic scale. R-value represents correlation coefficients. *p*-value was calculated by *t*-test.

Overexpression as well as depletion of ALC1 sensitizes cells to DNA-damaging agents [11], suggesting that maintaining a proper expression level of the cellular ALC1 protein is important for effective responses to DNA damage. We surveyed ~1,000 cancer-cell lines registered in the Cancer Cell Line Encyclopedia [44]. Surprisingly, the *ALC1* gene was amplified in a majority of the cell lines (Fig 6A), indicating that the *ALC1* locus may be extremely unstable and susceptible to gene amplification. The expression level of ALC1 significantly correlates with that of PARP1 (Fig 6B), but the molecular mechanisms underlying this correlation remain unclear. One possible scenario is that toxic effects caused by overexpression of ALC1 might be suppressed by simultaneous upregulation of PARP1. The correlation also supports the idea that appropriate control of ALC1 expression contributes to genome maintenance of malignant cells.

## Discussion

During the past decade, roles played by ALC1 in the regulation DNA repair as well as its PARP1 stimulated chromatin-repositioning enzyme activity have been well determined [6, 10, 11, 37, 45, 46]. In this study, we explored ALC1’s possible role in BER in the chicken DT40 and human TK6 cell lines. Alkaline-comet and alkaline-elusion assays consistently showed that ALC1 promotes BER a step after SSB formation in both chicken and human cells (Figs 2 and 3). These results were consistent with the previously suggested possible roles of ALC1 in DNA repair [6, 46]. We also demonstrated the epistatic relationship between *ALC1*^*-/-*^ and *PARP1*^*-/-*^ mutations in cellular resistance to H_2_O_2_ and MMS in chicken DT40 cells (Fig 1). The epistatic relationship between *ALC1*^*-/-*^ and *PARP1*^*-/-*^ is consistent with the previous reports showing that ALC1 is recruited to the site of DNA damage and activated through PARylation [6, 37, 45]. Since PARP1 plays a role in BER, this epistatic relationship further supports the conclusion that ALC1 plays a role in BER. We thus conclude that ALC1 significantly promotes BER.

The higher sensitivity to H_2_O_2_ found in *ALC1*^*-/-*^ DT40 cells compared to *PARP1*^*-/-*^ DT40 cells (Fig 1) reveals the critical role of ALC1 in cellular tolerance to oxidative DNA damage. This observation is very surprising, because the promotion of SSB repair by PARylation has previously been attributed solely to the enhanced recruitment of XRCC1 to DNA-damage sites by PARP. Our data reveal that ALC1 contributes to the PARP-dependent promotion of BER without affecting the recruitment of XRCC1 or Polβ to DNA lesions (Fig 4). We thus conclude that ALC1 plays a key role in SSB repair, independent of both XRCC1 and Polβ (Fig 4).

An important question is, how does ALC1 contribute to BER? A significant delay in the repair of SSBs, which are BER intermediates (Figs 2 and 3), indicates that ALC1 contributes to promoting the sealing of SSBs. ALC1 facilitates chromatin relaxation, most likely at DNA-damage sites (Fig 5). This is consistent with the fact that attenuation of ALC1’s ATPase activity by the E165Q mutation completely inactivates its functions (Fig 1) [6, 37]. One possible scenario is that chromatin remodeling by ALC1 might facilitate long-patch repair by removing nucleosomes for DNA-repair synthesis by DNA polymerases β and δ. Future research is indicated to clarify ALC1’s role in BER by conducting *in vitro* BER in a chromatin context.

## Supporting information

S1 Fig Generation of *ALC1*^-/-^ cells from chicken DT40 cells. (A) Schematic showing part of the *GdALC1* locus. The filled boxes represent exons. The thick lines show the genomic region amplified for the targeting-vector arms. The relevant *Sca*I and *Bam*HI sites and the position of the probe used for Southern blot analysis are indicated. (B) Southern blot analysis of *wild-type* (+/+), heterozygous mutant (+/-), and homozygous mutant (-/-). *Sca*I- and *Bam*HI-digested genomic DNA was hybridized with the probe shown in (A). (C) Representative cell-cycle distribution of the indicated cell cultures as measured by BrdU incorporation and DNA content in flow-cytometric analysis. The upper, lower left, lower right, and leftmost gates correspond to cells in the S, G1, and G2/M phases, and sub-G1 fraction, respectively. Numbers show the percentage of cells that fall within each gate. (D) Generation of the ALC1-ATPase-deficient *ALC1*^*-/E165Q*^ clone. Schematic showing part of the *GdALC1* locus. The filled boxes represent exons. The thick lines show the genomic region amplified for the targeting-vector arms. The relevant *Bam*HI site and the position of the probe used for Southern blot are indicated.
(PDF)

S2 Fig Disruption of *ALC1* gene in human TK6 cells. (A) Schematic of part of the human *ALC1* locus. The knockout constructs are shown below the locus. The filled boxes represent exons. The thick lines show the genomic region amplified for targeting-vector arms.
(B) *Wild-type* (+/+) as well as *ALC1*^-/-^ (-/-) TK6 cells were subjected to RT-PCR using *GAPDH*- or *ALC1*-specific primers.
(C) *Wild-type* (+/+) as well as *ALC1*^-/-^ (-/-) TK6 cells were subjected to western blot using α-ALC1 specific antibody. The blot was probed with α-βactin antibody as a loading control.
(PDF)

S3 Fig The accumulation of MMS-induced SSBs in the *ALC1*^-/-^ human TK6 cells. The average of the median of tail moments from 100 comets in each assay is displayed on the y-axis on a linear scale. Error bars represent standard deviations from three independent experiments. Indicated TK6 cells were treated with indicated concentrations of MMS at 37°C for 15 min. In this condition, base damage and repair occur in parallel.
(PDF)
