# Supplementary material for: ALC1/CHD1L, a chromatin-remodeling enzyme, is required for efficient base excision repair
Source: PLoS One. 2017 Nov 17;12(11):e0188320. doi: 10.1371/journal.pone.0188320 (PMC5693467; doi:10.1371/journal.pone.0188320)
Supplement: S1 Fig — (A) Schematic showing part of the GdALC1 locus. The filled boxes represent exons. The thick lines show the genomic region amplified for the targeting-vector arms. The relevant ScaI and BamHI sites and the position of the probe used for Southern blot analysis are indicated. (B) Southern blot analysis of wild-type (+/+), heterozygous mutant (+/-), and homozygous mutant (-/-). ScaI- and BamHI-digested genomic DNA was hybridized with the probe shown in (A). (C) Representative cell-cycle distribution of the indicated cell cultures as measured by BrdU incorporation and DNA content in flow-cytometric analysis. The upper, lower left, lower right, and leftmost gates correspond to cells in the S, G1, and G2/M phases, and sub-G1 fraction, respectively. Numbers show the percentage of cells that fall within each gate. (D) Generation of the ALC1-ATPase-deficient ALC1-/E165Q clone. Schematic showing part of the GdALC1 locus. The filled boxes represent exons. The thick lines show the genomic region amplified for the targeting-vector arms. The relevant BamHI site and the position of the probe used for Southern blot are indicated. (PDF) [file pone.0188320.s001.pdf]

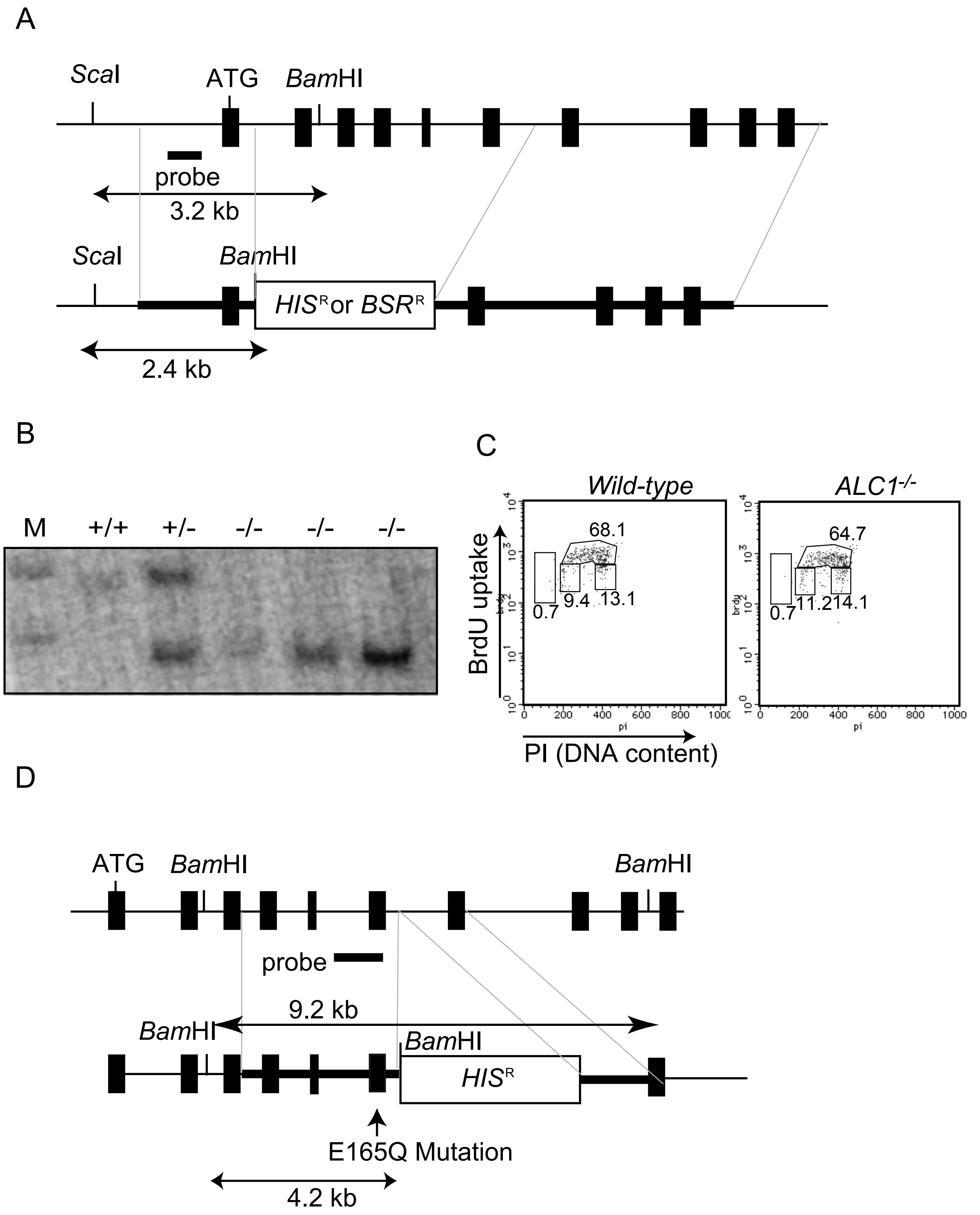

S1 Fig Generation of *ALC1* $^{-/-}$ cells from chicken DT40 cells. (A) Schematic showing part of the *GdALC1* locus. The filled boxes represent exons. The thick lines show the genomic region amplified for the targeting-vector arms. The relevant *Sca*I and *Bam*HI sites and the position of the probe used for Southern blot analysis are indicated. (B) Southern blot analysis of *wild-type* (+/+), heterozygous mutant (+/-), and homozygous mutant (-/-). *Sca*I- and *Bam*HI-digested genomic DNA was hybridized with the probe shown in (A). (C) Representative cell-cycle distribution of the indicated cell cultures as measured by BrdU incorporation and DNA content in flow-cytometric analysis. The upper, lower left, lower right, and leftmost gates correspond to cells in the S, G1, and G2/M phases, and sub-G1 fraction, respectively. Numbers show the percentage of cells that fall within each gate. (D) Generation of the ALC1-ATPase-deficient *ALC1-/E165Q* clone. Schematic showing part of the *GdALC1* locus. The filled boxes represent exons. The thick lines show the genomic region amplified for the targeting-vector arms. The relevant *Bam*HI site and the position of the probe used for Southern blot are indicated.
