# Supplementary material for: ALC1/CHD1L, a chromatin-remodeling enzyme, is required for efficient base excision repair
Source: PLoS One. 2017 Nov 17;12(11):e0188320. doi: 10.1371/journal.pone.0188320 (PMC5693467; doi:10.1371/journal.pone.0188320)
Supplement: S2 Fig — (A) Schematic of part of the human ALC1 locus. The knockout constructs are shown below the locus. The filled boxes represent exons. The thick lines show the genomic region amplified for targeting-vector arms. (B) Wild-type (+/+) as well as ALC1-/- (-/-) TK6 cells were subjected to RT-PCR using GAPDH- or ALC1-specific primers. (C) Wild-type (+/+) as well as ALC1-/- (-/-) TK6 cells were subjected to western blot using α-ALC1 specific antibody. The blot was probed with α-βactin antibody as a loading control. (PDF) [file pone.0188320.s002.pdf]

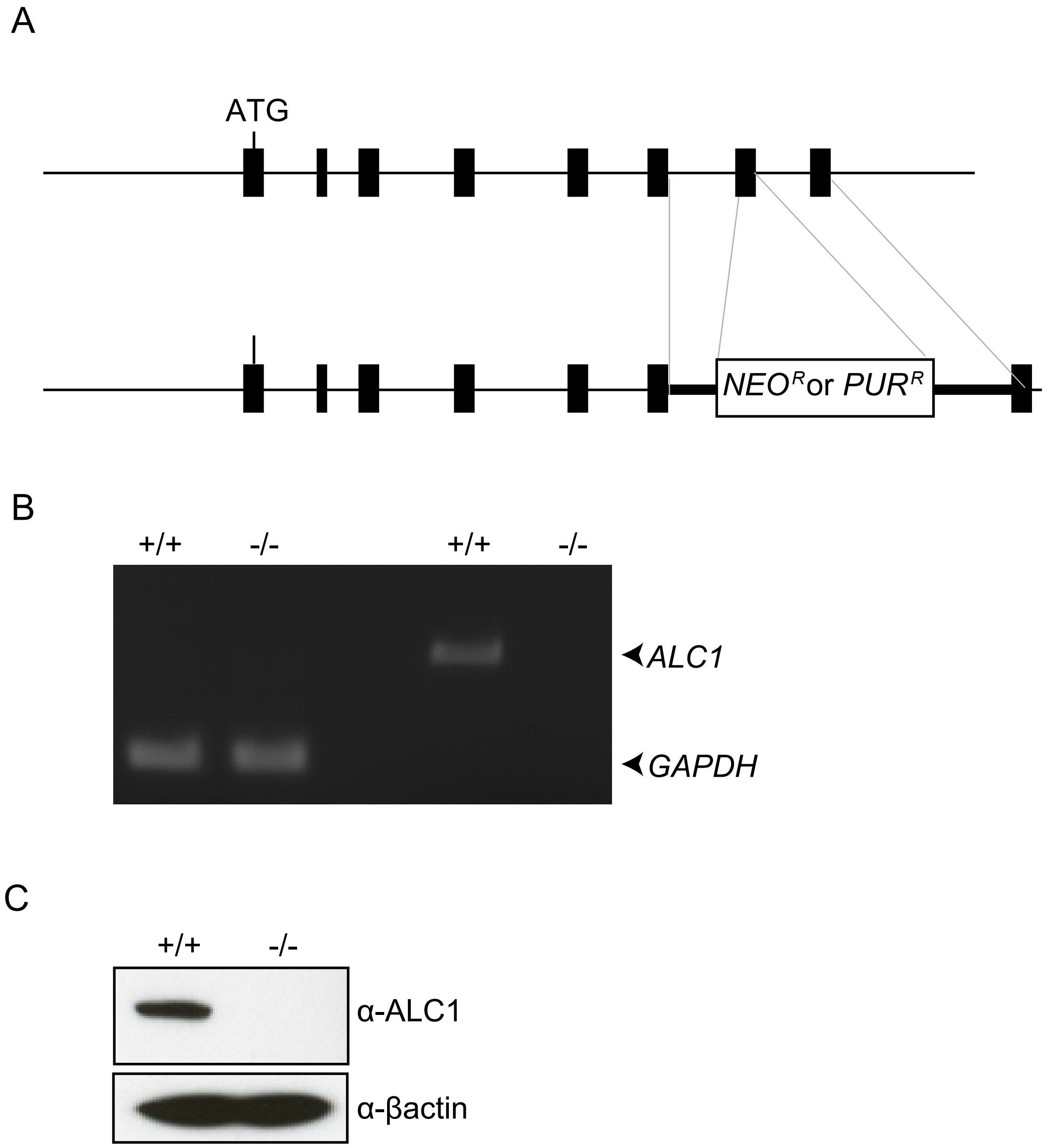

S2 Fig Disruption of *ALC1* gene in human TK6 cells.

(A) Schematic of part of the *hALC1* locus. The knockout constructs are shown below the locus. The filled boxes represent exons. The thick lines show the genomic region amplified for targeting-vector arms.

(B) *Wild-type (+/+)* as well as *ALC1*$^{-/-}$ (-/-) TK6 cells were subjected to RT-PCR using *GAPDH*- or *ALC1*-specific primers.

(C) *Wild-type (+/+)* as well as *ALC1*$^{-/-}$(-/-) TK6 cells were subjected to western blot using α-ALC1 specific antibody. The blot was probed with α-βactin antibody as a loading control.
