# Supplementary material for: ALC1/CHD1L, a chromatin-remodeling enzyme, is required for efficient base excision repair
Source: PLoS One. 2017 Nov 17;12(11):e0188320. doi: 10.1371/journal.pone.0188320 (PMC5693467; doi:10.1371/journal.pone.0188320)
Supplement: S3 Fig — The average of the median of tail moments from 100 comets in each assay is displayed on the y-axis on a linear scale. Error bars represent standard deviations from three independent experiments. Indicated TK6 cells were treated with indicated concentrations of MMS at 37°C for 15 min. In this condition, base damage and repair occur in parallel. (PDF) [file pone.0188320.s003.pdf]

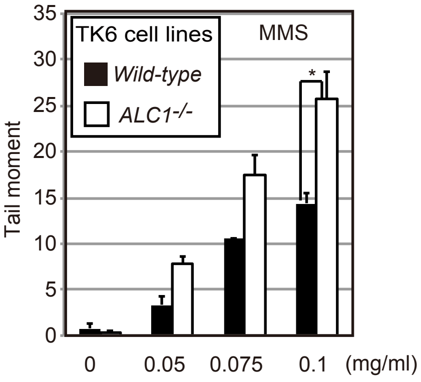

S3 Fig The accumulation of MMS-induced SSBs in the *ALC1*$^{-/-}$ human TK6 cells.

The average of the median of tail moments from 100 comets in each assay is displayed on the y-axis on a linear scale. Error bars represent standard deviations from three independent experiments. Indicated TK6 cells were treated with indicated concentrations of MMS at 37° C for 15 min. In this condition, base damage and repair occur in parallel.
